# Terrain-aware semantic mapping for cooperative subterranean exploration

**DOI:** 10.3389/frobt.2023.1249586

**Published:** 2023-10-03

**Authors:** Michael J. Miles, Harel Biggie, Christoffer Heckman

**Affiliations:** ^1^ Department of Mechanical Engineering, University of Colorado Boulder, Boulder, CO, United States; ^2^ Department of Computer Science, University of Colorado Boulder, Boulder, CO, United States

**Keywords:** subterranean environments, field robots, semantic grid mapping, terrain mapping, cooperative exploration, DARPA subterranean challenge

## Abstract

Navigation over torturous terrain such as those in natural subterranean environments presents a significant challenge to field robots. The diversity of hazards, from large boulders to muddy or even partially submerged Earth, eludes complete definition. The challenge is amplified if the presence and nature of these hazards must be shared among multiple agents that are operating in the same space. Furthermore, highly efficient mapping and robust navigation solutions are absolutely critical to operations such as semi-autonomous search and rescue. We propose an efficient and modular framework for semantic grid mapping of subterranean environments. Our approach encodes occupancy and traversability information, as well as the presence of stairways, into a grid map that is distributed amongst a robot fleet despite bandwidth constraints. We demonstrate that the mapping method enables safe and enduring exploration of subterranean environments. The performance of the system is showcased in high-fidelity simulations, physical experiments, and Team MARBLE’s entry in the DARPA Subterranean Challenge which received third place.

## 1 Introduction

Robots have proliferated in structured and well-controlled environments such as those in warehousing and retail due to the simplicity and consistency of such environments. However, unstructured and subterranean environments remain a challenge for most autonomous robots due to their complex topology, large size, and lack of infrastructure or available prior knowledge of the environment. Such environments are especially challenging for mobile, ground-based robots, which must contend with rugged terrain (i.e., obstacle-laden indoor environments, rough or off-road outdoor environments) during their operation. The complexity of this problem is further exacerbated in high-risk scenarios, e.g., autonomous search and rescue operations, in which a team of robots must quickly and continuously explore such environments with limited inter-robot communication abilities. Thus, the mapping solution used by ground robots to understand their environments is critical to the task of autonomous, multi-agent exploration. An effective mapping solution for such scenarios should generate high-fidelity reconstructions of the environment which include information that may aid in the operation, such as terrain features and objects or structures of interest. Furthermore, the solution should facilitate rapid construction and efficient sharing to enable collaborative exploration.

Early approaches to robot mapping focused primarily on occupancy grid mapping, in which an environment is discretized into a 2D (or later, 3D) grid. Each grid cell independently models the probability that the area/volume to which it corresponds consists of free or occupied space. These approaches generally model occupancy by integrating range and bearing measurements over time via a binary Bayes filter. One of the first of these approaches was that of Moravec and Elfes (1985) which utilizes an array of twenty-four sonar transducers covering 360° around a mobile robot to generate a 2D occupancy grid map. Payeur et al. (1997) makes the extension of occupancy grid maps from 2D to 3D computationally tractable by introducing a closed-form approximation of the occupancy probability function and storing the cells in a multi-resolution octree. Despite their early beginnings, occupancy grid mapping approaches remain at the forefront of many modern robot mapping solutions. Hornung et al., 2013 introduces OctoMap, an extremely popular (Lluvia et al., 2021) open-source mapping package that features an octree-based data structure and lossless compression for highly efficient occupancy grid mapping.

Occupancy grid maps are efficient at mapping the general occupancy state of an environment but do not scale well for high-resolution mapping of surfaces, which are of particular interest in terrain traversability mapping. Oleynikova et al. (2017) introduces Voxblox which utilizes a Truncated Signed Distance Field (TSDF) -based map representation and voxel hashing for fast queries, however, this approach uses large amounts of memory, which restricts sharing with other robots in the fleet, especially for large environments. Shan et al. (2018) leverages probabilistic inference to build terrain maps from sparse lidar data by modeling traversability as a Bernoulli-distributed random variable, however, this approach requires training, which can become inaccurate in unknown environments. Labbé and Michaud (2019) presents RTAB-Map which maintains pointcloud maps via a memory-efficient loop closure approach, however, memory inefficiency in mapping still makes this approach prohibitive for sharing dense information.

Some approaches combine the simplicity of grid mapping with the height resolution of surface maps to yield 2.5D elevation maps, wherein the value of each horizontal grid cell corresponds to the probabilistic height of the surface at that location. Hebert et al. (1989) presents an early such approach that fused measurements from a scanning lidar imager into an elevation map for mapping potential footholds for a quadrupedal robot. Fankhauser et al., 2014; Fankhauser and Hutter 2016 extends this idea with GridMap, an open-source mapping package that generates multi-layer elevation and traversability maps in a robot-centric approach for a quadrupedal robot. Hines et al. (2021) utilizes GridMap for traversability-mapping in conjunction with an occupancy grid map which features novel “virtual surfaces” to encourage safe exploration amidst negative obstacles as part of Team CSIRO Data61’s entry in the DARPA Subterranean Challenge. Fan et al. (2021) also utilizes a similar elevation map in conjunction with a Conditional-Value-at-Risk metric for traversability-mapping as part of Team CoSTAR’s entry in the DARPA Subterranean Challenge. Haddeler et al. (2022) presents a method similar to our own which classifies semantic traversability from geometric pointcloud features and incorporated the information into a 2D elevation map.

In addition to traversability, the classification and mapping of objects or structures of interest, such as stairways, is also highly valuable. This is particularly important in the context of urban search and rescue where the ability to identify and navigate stairways is a critical feature of autonomous rescue systems. Harms et al. (2015) presents an approach to stair detection which utilized a stereo-vision camera system and a convolutional kernel to extract the alternating concave and convex edges of the steps of a stairway. Sánchez-Rojas et al. (2021) presents a hybrid visual and geometric approach to stair detection as well as a fuzzy logic controller for the alignment of robots to the detected stairway. (Westfechtel et al., 2016, Westfechtel et al., 2018) introduces StairwayDetection, an open-source package that directly classifies the various parts of stairways using a graph-based approach, including the stair tread, riser, and railing segments.

In this work, we present a terrain-mapping system based on semantically-encoded 3D grid maps. In addition to occupancy, the traversability of the terrain as well as the presence of stairways are geometrically classified and probabilistically mapped. Furthermore, a low-bandwidth method for sharing these maps amongst a heterogeneous fleet of quadrupedal and wheeled vehicles is shown. This mapping system was used in conjunction with a terrain-aware path planner as part of Team MARBLE’s entry into the DARPA Subterranean Challenge in which they took third place (Biggie et al., 2023). This opportunity provided an extremely unique and realistic environment in which to field deploy and validate the proposed terrain-mapping system. We hypothesize that the terrain-mapping system described herein, which is made possible with several novel and open-source subsystems in elegant coordination, constitutes an effective method to enable safe and enduring exploration in large, unstructured, subterranean environments.

## 2 Materials and methods

Briefly, the terrain-mapping system, called *MARBLE Mapping*, operates as follows. Classifications of terrain traversability and the presence of stairways are performed independently on unlabeled 3D pointclouds from geometric features and the resultant labeled pointclouds are spatially and temporally integrated into a 3D grid map. A path planner uses this semantic grid map to evaluate candidate plans for compatibility with the given platform and explore the subterranean environment. As the grid map is updated, differential maps containing compressed occupancy, traversability, and stair-presence information are queued and shared with other robot agents whenever a communication link is established. Upon receiving differential maps from external agents, these maps are fused into the recipient agent’s local grid map. Furthermore, a powerful lidar-inertial odometry solution called LIO-SAM (Shan et al., 2020) is used for online localization. The system operates on the Robot Operating System (ROS1) message-passing framework (Quigley et al., 2009) for internal communication and a custom UDP-based transport layer (Biggie and McGuire, 2022) for multi-agent communication.

**FIGURE 1:**
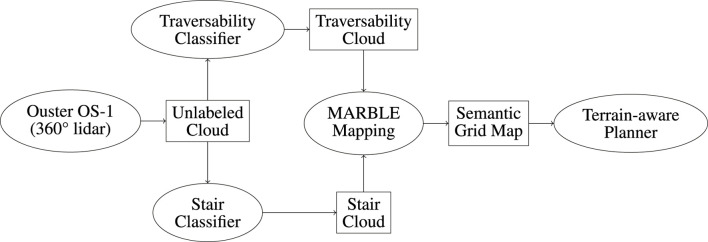
Overview of Team MARBLE’s terrain-aware navigation solution. Ellipses denote software packages. Rectangles denote data products.

While this work will be primarily concerned with the classification and mapping of traversability and stairways, differential map sharing and terrain-aware path planning are major considerations and will be used to evaluate the mapping method described here. The proposed method and its context in Team MARBLE’s terrain-aware navigation solution is illustrated in Figure 1.

### 2.1 Occupancy classification and mapping

Our semantic occupancy mapping pipeline is based on the framework provided in Hornung et al. (2013). Utilizing a time-of-flight lidar, this framework leverages pointcloud data to construct a 3D voxel-based representation of the surrounding environment. Points in the pointcloud constitute occupied space and the space between the sensor and the point is considered free. These occupancy classifications are probabilistically fused into the grid map.

The framework scales incredibly well due to the underlying octree data structure which provides efficient data storage and queries. An octree is a hierarchical structure for storing volumetric data contained in a 3D grid. Each volumetric cell (“voxel”) in the grid is represented by a node of the octree and can be recursively combined to represent coarser volumes of decreased resolution (“pruned”) or recursively subdivided to represent finer volumes of increased resolution (“expanded”). The primary benefit of the octree data structure is that it does not need to maintain the position of a voxel or its size explicitly. Instead, this information is reconstructed from inexpensive traversal of the octree, which results in a highly memory-efficient map. This low-memory representation is fundamental to sharing these maps between robot agents via a low-bandwidth communication network.

Occupancy grids suffer from uncertainty arising from several sources: sensor noise, localization drift, misalignment between multiple sensors and/or robots, etc. This motivates the use of a probabilistic occupancy representation to classify the occupancy state of an environment; in this work, we use the binary Bayes filter (Eqs. 5 and 6) as was done in (Moravec and Elfes 1985; Thrun 2002).

The posterior probability *P*_*occ*_(*v*|*z*_*occ,*1*:t*_) that some voxel *v* is occupied, given sensor measurements *z*_*occ,*1*:t*_ is estimated via:

$$\begin{array}{cc} & P_{\mathrm{occ}}\left(v|z_{\mathrm{occ,}1\mathrm{:t}}\right)\\ & \;=\;{\left[1+\frac{1-P_{\mathrm{occ}}\left(v|z_{\mathrm{occ,t}}\right)}{P_{\mathrm{occ}}\left(v|z_{\mathrm{occ,t}}\right)}\frac{1-P_{\mathrm{occ}}\left(v|z_{\mathrm{occ,}1\mathrm{:t}-1}\right)}{P_{\mathrm{occ}}\left(v|z_{\mathrm{occ,}1\mathrm{:t}-1}\right)}\frac{P_{\mathrm{occ}}\left(v\right)}{1-P_{\mathrm{occ}}\left(v\right)}\right]}^{-1}\text{ ,}\\ & \;\text{where }\;P_{\mathrm{occ}}\left(v|z_{\mathrm{occ,}1\mathrm{:t}}\right)\in \left(0,1\right)\;. \end{array}$$ (1)

A voxel with an occupancy probability of greater than the prior *P*_*occ*_(*v*) is considered occupied, and a voxel is considered free space if it has an occupancy probability of less than the prior. An occupancy probability equal to the prior implicitly defines a voxel whose occupancy status is unknown.

Equation 1 is rewritten in log-odds form to avoid numerical instabilities and quicken updates *L*_*occ*_(*v*|*z*_*occ,t*_) to the current log-odds occupancy estimate *L*_*occ*_(*v*|*z*_*occ,*1:*t*_):

$$L_{\mathrm{occ}}\left(v|z_{\mathrm{occ,}1\mathrm{:t}}\right)=\;\text{log}\left(\frac{P_{\mathrm{occ}}\left(v|z_{\mathrm{occ,}1\mathrm{:t}}\right)}{1-P_{\mathrm{occ}}\left(v|z_{\mathrm{occ,}1\mathrm{:t}}\right)}\right)$$ (2a)

$$=\;L_{\mathrm{occ}}\left(v|z_{\mathrm{occ,}1\mathrm{:t}-1}\right)+L_{\mathrm{occ}}\left(v|z_{\mathrm{occ,t}}\right)\;,$$ (2b)

$$\begin{array}{ll} \;\text{where} & \;L_{\mathrm{occ}}\left(v|z_{\mathrm{occ,}1\mathrm{:t}}\right)\in \left[L_{\mathrm{occ,min}},L_{\mathrm{occ,max}}\right]\;,\\ & \;L_{\mathrm{occ,min}}\in \left(-\infty ,0\right)\text{ , }L_{\mathrm{occ,max}}\in \left(0,\infty \right)\;,\\ & \;L_{\mathrm{occ}}\left(v|z_{\mathrm{occ,t}}\right)=\left\{\begin{array}{c} L_{\mathrm{occ,hit}}>0\text{ on occupied space}\;\\ L_{\mathrm{occ,miss}}<0\text{ on free space}. \end{array}\right. \end{array}$$

The updates are represented by the log-odds form of the inverse sensor model *P*_*occ*_(*v*|*z*_*occ,t*_). For a beam-based range finder (e.g., lidar, sonar), the inverse sensor model can be approximated as a constant in log-odds form (Thrun, 2002). A ray is cast from the sensor to the incident voxel and all voxels that the beam passes through, as determined by the Bresenham algorithm (Amanatides and Woo, 1987), are measured as free with a log-odds update of $L_{\mathrm{occ,miss}}$ , while the incident voxel is measured as occupied and updated with $L_{\mathrm{occ,hit}}$ . The log-odds occupancy estimate is also bounded between $L_{\mathrm{occ,min}}$ and $L_{\mathrm{occ,max}}$ to improve performance in dynamic environments and increase memory efficiency (via pruning).

### 2.2 Traversability classification and mapping

We extend Hornung et al. (2013) to include traversability classification and mapping capabilities, thus enabling traversability-aware navigation and encouraging robot endurance in the presence of rough terrain. Specifically, we estimate a continuous *traversability cost*, i.e., the difficulty of traversing the terrain, primarily as it pertains to wheeled or tracked robots.

The traversability cost estimation described here relies on the pointcloud’s geometric attributes, including slope and surface curvature. Other geometric characteristics, like roughness (fitness to a plane), have been shown to improve navigation performance (Ye, 2007). The incorporation of non-geometric features, such as friction (Hughes et al., 2019; Rodríguez-Martínez et al., 2019) and collapsibility (Haddeler et al., 2022), may also significantly improve navigation performance over adverse terrain, however, these features are often computationally expensive to calculate and less applicable to travel at low-moderate speeds. Learned terrain features (Guan et al., 2022; Shaban et al., 2022; Seo et al., 2023) offer a cheap and fast approach for real-time traversability estimation, yet the efficacy of these methods remains dependent on costly training and poses challenges in adapting to completely unknown environments. After extensive physical and simulated testing, we chose to focus solely on the slope and surface curvature as this approach proved to be efficient and provided adequate assurance of the robot’s endurance.

We use the same 3D pointcloud from a time-of-flight lidar as was used in Section 2.1 to classify traversability in the environment (Figure 3A). The proposed method estimates the instantaneous traversability cost *τ*_*p*,*t*_ of a point *p* at time *t* (Equation 3) as a normalized weighted sum of the slope $\left(1-|\hat{n}\cdot \hat{k}|\right)$ and the curvature *κ* at the point:

$$\tau_{p,t}=\|c_{\mathrm{slope}}{\left(1-|{\hat{n}}_{p,t}\cdot \hat{k}|\right)}^{3}+c_{\mathrm{curv}}\kappa_{p,t}\|\in \left[0,1\right],$$ (3)

where a traversability cost of *τ*_*p*,*t*_ = 0 is easily traversed and *τ*_*p*,*t*_ = 1 is untraversable. The slope penalty *c*_*slope*_ and curvature penalty *c*_*curv*_ were tuned over numerous physical and simulated test deployments to prevent navigation into obstacles or up steep faces, and to encourage routes over smooth terrain, respectively. The slope, curvature, and final traversability cost as applied to the entire pointcloud can be seen in Figures 3B–D, respectively.

In our implementation, we pre-process the pointcloud with a voxel-grid filter to achieve a uniform resolution of 5 cm and then estimate the normal vector and surface curvature of each point via a least-squares plane fit to its twenty-four nearest neighbors as determined by a kd-tree. Applied to a relatively planar patch of points, this approach effectively estimates normal information from a square patch covering 400 square centimeters. This approach is faster but yields similar results compared to pre-processing the full-resolution pointcloud and estimating normal information from neighbors based on distance directly.

Because slope and curvature represent the first and second derivatives of the positions of the points, respectively, they are jointly afflicted by the uncertainty associated with the point positions. We empirically analyze the effect of point position noise on the point traversability cost by generating and calculating the traversability cost terms associated with a noisy, planar pointcloud (Table 1). We generate pointclouds for four sets of parameters with varying amounts of noise in the x, y, and *z*-axes. Then we calculate the slope and curvature cost terms in Eq. 3 and analyze the average and standard deviation cost of each point in the pointcloud over 20 samples of each parameter set. The practical effect is that extremely noisy pointclouds of planar terrain may appear especially rough or slightly sloped, as illustrated in Table 1. We wish to avoid extremely rough terrain as equally as sloped terrain and we perform instantaneous traversability classification of single pointclouds using a high precision 3D lidar which exhibits sub-centimeter standard deviation in point position (Ouster, 2023), so the effects of noise are negligible in our approach.

**TABLE 1:** Noise analysis of a planar pointcloud, as in Figure 2. Four sets of parameters describing the standard deviation of the points in meters in the *x*, *y*, and *z*-axes are used: **A** (0.0, 0.0, 0.0), **B** (1.0, 1.0, 0.0), **C** (1.0, 1.0, 0.10), and **D** (1.0, 1.0, 0.5). The mean and standard deviation of the slope cost and curvature cost terms of Eq. 3 in each pointcloud over 20 samples of each parameter set are shown. Throughout this work, we use a slope gain *c*_*slope*_ of 20.0 and a curvature gain *c*_*curv*_ of 2.0.

Average traversability cost by point position noise				
Cost term	A	B	C	D
Slope	0.0 ± 0.0	0.0 ± 0.0	0.0 ± 0.0	2.254 ± 0.029
Curvature	0.0 ± 0.0	0.0 ± 0.0	0.097 ± 0.005	0.364 ± 0.001

**FIGURE 2:**
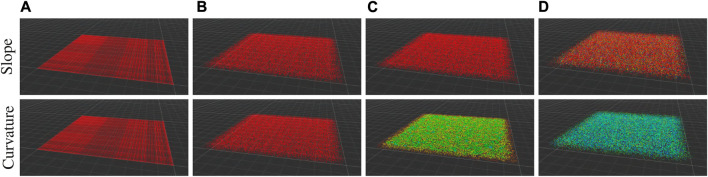
A typical pointcloud generated for the noise analysis in Table 1 for each parameter set **(A–D)**. The pointcloud has a side length of 10 m and a horizontal resolution of 5 cm. The point colors represent the cost terms of Eq. 3: the slope cost term on the top row, where red points indicate a cost of 0.0 and blue points indicate a curvature cost of 20.0; and the curvature cost term on the bottom row, where red points indicate a cost of 0.0 and blue points indicate a curvature cost of 0.7.

**FIGURE 3:**
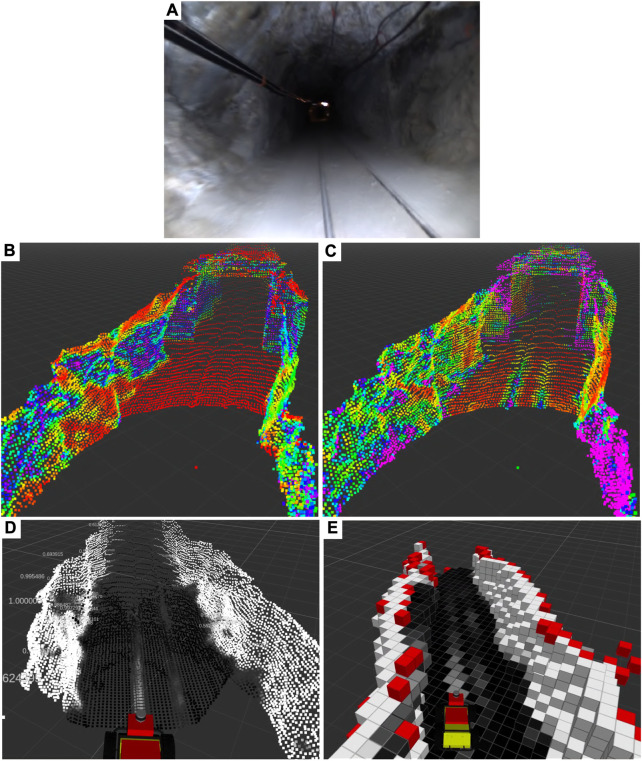
A traversability map is generated during a physical test deployment in the Edgar Mine in Idaho Springs, CO, USA **(A)**. Geometric measurements on 3D pointclouds, including normal **(B)** and curvature **(C)** estimation, are combined to produce traversability-labeled pointclouds **(D)** which are then fused into a grid map **(E)**. Note in **(E)** the classification of the walls as non-traversable (white), the rail tracks as semi-traversable (grey), and the surrounding flat ground as traversable (black). Red voxels indicate those which are occupied but have no traversability data.

We estimate the traversability cost *τ*_*v*,*t*_ of the region within a given voxel *v* of the grid map from points that correspond to that voxel p∈V . The Figure 2 traversability-labeled pointclouds are spatially and temporally fused into a semantic grid map (Figure 3E) via an exponential moving average:

$$\tau_{v,t}=\tau_{v,t-1}P_{\mathrm{occ,v,t}}+\tau_{p\in V,t}\left(1-P_{\mathrm{occ,v,t}}\right)\in \left[0,1\right]\;.$$ (4)

This approach incorporates new measurements more readily when the probability of occupancy is low, as these points contain more information, and this method performed best during testing.

### 2.3 Stairway classification and mapping

While a geometric assessment of the environment prevents the robot from traversing over unsafe areas, there are situations where we want to exploit semantic information to explicitly traverse over known obstacles such as a stairway. To this end, we utilize the aforementioned unlabeled 3D pointcloud to classify stairways in the environment based on the work in Westfechtel et al., 2016, Westfechtel et al., 2018. We utilize this package in the proposed work for its superior performance in detecting various types of stairways from sparse pointcloud data. This package was modified to operate in the ROS framework.

Briefly, classification via this approach consists of four major steps: 1) preanalysis, in which downsampling, filtering, normal/curvature estimation, and floor separation are performed; 2) segmentation via region growing algorithm, which segments the pointcloud into continuous regions; 3) plane extraction, in which the flat planes which constitute the riser, tread, and rail regions of each step are extracted; and 4) recognition, in which the tread and riser regions are connected and analyzed via a graph to determine whether they make up a valid set of stairs.

**FIGURE 4:**
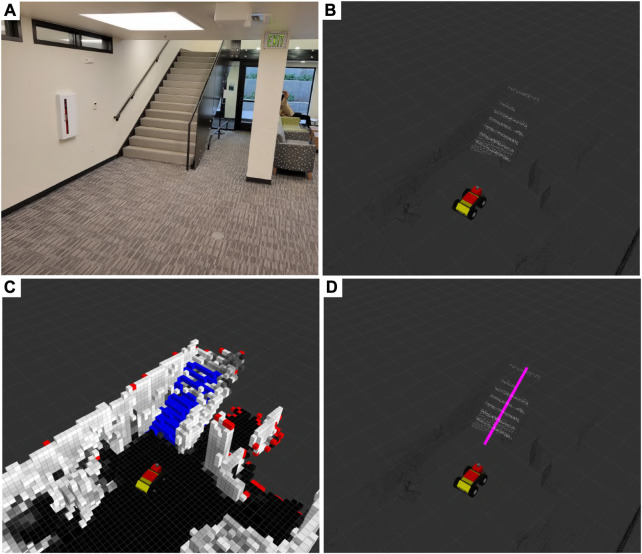
Stairways **(A)** are mapped during a physical test deployment in the Engineering Center at the University of Colorado Boulder in Boulder, CO, United States of America. Parts of a 3D pointcloud that belong to a stairway white in **(B)** are segmented via StairwayDetector (Westfechtel et al., 2016; 2018) and then fused into an occupancy map **(C)**. Additionally, we cluster stair voxels (blue) and extract the primary axis pink in **(D)** to inform the terrain-aware planner.

Similarly to the case of occupancy data in Section 2.1, we use the binary Bayes filter (Equations 5 and 6) to integrate the stair-labeled pointclouds (Figure 4B) into the semantic grid map (Figure 4C). We model the probability *P*_*stair*_(*v*|*z*_*stair,*1*:t*_) that a voxel *v* is spatially correlated with a stairway given measurements *z*_*stair,*1*:t*_ as points in a labeled pointcloud produced by a binary stairway classifier up until time *t*:

$$\begin{array}{cc} & P_{\mathrm{stair}}\left(v|z_{\mathrm{stair,}1\mathrm{:t}}\right)\\ & \;=\;{\left[1+\frac{1-P_{\mathrm{stair}}\left(v|z_{\mathrm{stair,t}}\right)}{P_{\mathrm{stair}}\left(v|z_{\mathrm{stair,t}}\right)}\frac{1-P_{\mathrm{stair}}\left(v|z_{\mathrm{stair,}1\mathrm{:t}-1}\right)}{P_{\mathrm{stair}}\left(v|z_{\mathrm{stair,}1\mathrm{:t}-1}\right)}\frac{P_{\mathrm{stair}}\left(v\right)}{1-P_{\mathrm{stair}}\left(v\right)}\right]}^{-1}\text{ ,}\\ & \;\text{where }\;P_{\mathrm{stair}}\left(v|z_{\mathrm{stair,}1\mathrm{:t}}\right)\in \left(0,1\right)\;; \end{array}$$ (5)

$$L_{\mathrm{stair}}\left(v|z_{\mathrm{stair,}1\mathrm{:t}}\right)=\;\text{log}\left(\frac{P_{\mathrm{stair}}\left(v|z_{\mathrm{stair,}1\mathrm{:t}}\right)}{1-P_{\mathrm{stair}}\left(v|z_{\mathrm{stair,}1\mathrm{:t}}\right)}\right)$$ (6a)

$$=\;L_{\mathrm{stair}}\left(v|z_{\mathrm{stair,}1\mathrm{:t}-1}\right)+L_{\mathrm{stair}}\left(v|z_{\mathrm{stair,t}}\right)\;,$$ (6b)

$$\begin{array}{ll} \;\text{where} & \;L_{\mathrm{stair}}\left(v|z_{\mathrm{stair}}\right)\in \left[L_{\mathrm{stair,min}},L_{\mathrm{stair,max}}\right]\;,\\ & \;L_{\mathrm{stair,min}}\in \left(-\infty ,0\right)\text{ , }L_{\mathrm{stair,max}}\in \left(0,\infty \right)\;,\\ & \;L_{\mathrm{stair}}\left(v|z_{\mathrm{stair,t}}\right)=\left\{\begin{array}{c} L_{\mathrm{stair,hit}}>0\text{ on stair}\;\\ L_{\mathrm{stair,miss}}<0\text{ on non}-\text{stair}. \end{array}\right. \end{array}$$

The parameters of the algorithm are tuned so as to heavily weigh positive stair-point detections and lightly weigh negative detections. This aggressive strategy was adopted based on observations of sparse true positive detections but very few false positive detections and to encourage comprehensive exploration by stair-capable platforms.

In addition to the semantic map itself, the principal axis of the stairway (seen in Figure 4D) and a Boolean indicator as to whether the robot is nearing the start of a stairway is calculated and published for use by the navigation system. The principal axis of the stairway is calculated by clustering the stair voxels and performing eigenvalue decomposition on the cluster. This provides a straight-line path that the navigation system can use to traverse the stairway in a quick and orderly manner. The Boolean indicator serves as a signal to the low-level vehicle controller to transition from a non-stair-traversing state to a stair-traversing state and *vice versa*.

### 2.4 Differential map merging and sharing

Traversing large subterranean environments efficiently with multiple agents can be achieved using a shared semantic representation of the environment. However, regularly transmitting large, probabilistic, high-fidelity maps of such large environments over bandwidth-constrained wireless networks is not practical. Map differences are an intuitive solution that has been shown to facilitate efficient data transfers (Sheng et al., 2004).

**FIGURE 5:**
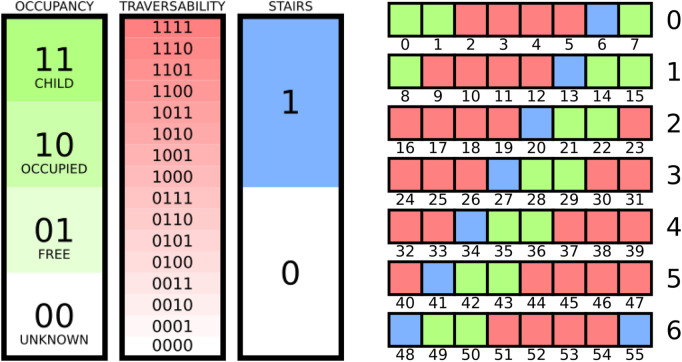
Compression scheme for encoding the semantic grid map. Occupancy information requires 2 bits, traversability information requires 4 bits, and stair-presence information requires 1 bit, for a total of 7 bits per voxel (left). All eight sub-cubes of a child node of the octree require 6 bytes (right).

Prior to sharing, the system compresses its locally-maintained, fully-probabilistic 3D grid map into a memory-efficient form. Compression involves binning the data associated with each voxel into a reduced set of classifications while preserving expressive information. An illustration of the compression scheme can be seen in Figure 5. The octree data structure also enables maps to be “pruned” on send and “expanded” on receipt to further improve network efficiency.

Occupancy information in the grid map is compressed similarly to that of the original implementation in Hornung et al. (2013):

$$B_{\mathrm{occ}}\left(n\right)=\left\{\begin{array}{cc} B_{\mathrm{occupied}}\; & \;\text{if}\;isLeaf\left(\right)\;\text{and}\;P_{\mathrm{occ}}\left(v|z_{\mathrm{occ,}\mathrm{1:}t}\right)>P_{\mathrm{occ}}\left(v\right)\\ B_{\mathrm{free}}\; & \;\text{if}\;isLeaf\left(\right)\;\text{and}\;P_{\mathrm{occ}}\left(v|z_{\mathrm{occ,}\mathrm{1:}t}\right)<P_{\mathrm{occ}}\left(v\right)\\ B_{\mathrm{unknown}}\; & \;\text{if}\;isLeaf\left(\right)\;\text{and}\;P_{\mathrm{occ}}\left(v|z_{\mathrm{occ,}\mathrm{1:}t}\right)=P_{\mathrm{occ}}\left(v\right),\\ B_{\mathrm{child}}\; & \;\text{else} \end{array}\right.\;$$ (7)

where $B_{\mathrm{occ}}\left(n\right)\in Z_{4}$ is the occupancy classification of node *n* in the octree, *isLeaf*() is true if the node is a “leaf” node (i.e., a node at the greatest depth of the octree and for which a corresponding voxel *v* exists), $B_{\mathrm{occupied}}$ is a classification which defines a leaf node as an occupied voxel, $B_{\mathrm{free}}$ is a classification which defines a leaf node as a free voxel, $B_{\mathrm{unknown}}$ is a classification which defines a leaf node as a voxel with unknown occupancy, $B_{\mathrm{child}}$ is a classification which defines the node as a non-leaf node that requires further expansion, *P*_*occ*_(*v*|*z*_*occ,*1*:t*_) ∈ (0, 1) is the estimated probability that the voxel is occupied after measurements *z*_*occ,*1*:t*_, and *P*_*occ*_(*v*) ∈ (0, 1) is the prior probability that the voxel is occupied.

The traversability measurement of each voxel is compressed via discretization to enable expressiveness in the traversability information in addition to bandwidth efficiency. The measurement consumes *m*-bits of memory per voxel to represent 2^*m*^ traversability classifications, such that

$$B_{\mathrm{trav}}\left(v\right)=B_{\mathrm{trav,j}}\text{ for }P_{\mathrm{trav,j-}1}<P_{\mathrm{trav}}\left(v|z_{\mathrm{trav,}\mathrm{1:}t}\right)\leq P_{\mathrm{trav,j}},$$ (8)

where $B_{\mathrm{trav}}\left(v\right)\in Z_{2^{m}}$ is the discretized traversability classification of voxel *v* and $B_{\mathrm{trav,j}}$ is the *j*th classification representing probabilities between $P_{\mathrm{trav,j-}1}\left(v\right)$ and $P_{\mathrm{trav,j}}\left(v\right)$ for *j* = 1, …, *m*. Throughout this work, *m* = 4.

Stair-presence information in the grid map is compressed via a simple binary encoding:

$$B_{\mathrm{stair}}\left(v\right)=\left\{\begin{array}{cc} B_{\mathrm{stair}}\; & \;\text{for}\;P_{\mathrm{stair}}\left(v|z_{\mathrm{stair,}1\mathrm{:t}}\right)>P_{\mathrm{stair}}\left(v\right)\\ B_{\mathrm{nonstair}}\; & \;\text{for}\;P_{\mathrm{stair}}\left(v|z_{\mathrm{stair,}1\mathrm{:t}}\right)\leq P_{\mathrm{stair}}\left(v\right), \end{array}\right.$$ (9)

where $B_{\mathrm{stair}}\left(v\right)\in Z_{2}$ is the occupancy classification of voxel *v*, $B_{\mathrm{stair}}$ is a classification which defines a voxel as corresponding to a stairway, $B_{\mathrm{nonstair}}$ is a classification which defines a voxel as not corresponding to a stairway, *P*_*stair*_(*v*|*z*_*stair,*1*:t*_) ∈ (0, 1) is the estimated probability that the voxel corresponds to a stairway after measurements *z*_*stair,*1*:t*_, and *P*_*stair*_(*v*) ∈ (0, 1) is the prior probability that the voxel corresponds to a stairway.

Instead of expensively sharing whole compressed grid maps, we developed a differential mapping and merging solution to reduce network bandwidth. In our approach, each agent generates two semantic grid maps: a “self-map” (Eq. 10a) and based solely on the aggregation of its own measurements; and a “merge-map” (Eq. 10c) and based on measurements from the whole fleet. Newly updated voxels of the self-map are extracted over predetermined time intervals and constructed into a “diff-map” (Eq. 10b). The diff-maps are transmitted to other agents as network conditions allow and are incorporated into their merge-maps. Each agent gives priority to its own measurements and incorporates only those voxels from external diff-maps into its merge-map that correspond to “unknown” voxels in its self-map. This strategy ensures that the merge-map remains uncontaminated by misaligned or corrupted diff-maps sent by compromised agents, thereby eliminating the necessity for cooperative localization while still enabling cooperative exploration. Formally.

$$S_{i,t}=F\left(v\right)\;\forall \;v\in V\;\text{if}\;P_{\mathrm{occ}}\left(v|z_{\mathrm{occ,}1\mathrm{:t}}\right)\neq P_{\mathrm{occ}}\left(v\right)$$ (10a)

$$D_{i,t}=S_{i,t}-\cap \left(S_{i,t-1},S_{i,t}\right)$$ (10b)

$$M_{j,t}=\cup \left(S_{j,t},D_{i,t}-\cap \left(S_{j,t},D_{i,t}\right)\right),$$ (10c)

Where *S*_*i*,*t*_ is the self-map of agent *i* at time *t*, F is a nonlinear aggregation function, V is the set of all observed voxels, *P*_*occ*_(*v*|*z*_*occ,*1*:t*_) is the probability that voxel *v* is occupied given measurements *z*_*occ,*1*:t*_, *P*_*occ*_(*v*) is the prior probability that voxel *v* is occupied, *D*_*i*,*t*_ is the diff-map of agent *i* at time *t*, and *M*_*j*,*t*_ is the merge-map of agent *j* at time *t* (assuming the diff-map is instantaneously transmitted from agent *i* to agent *j*).

Transmission of diff-maps between agents is performed over a mesh network of radio beacons with a custom transport layer which facilitates fast reconnect times and prioritization controls (Biggie and McGuire, 2022).

### 2.5 Terrain-aware, cooperative path planning

A sampling-based path planner (Ahmad and Humbert, 2022) utilizes the mapping method described thus far to quickly and safely explore large, unstructured environments amidst rough terrain and stairs. The planner maintains a graph of nodes over the environment and searches the graph via A* for paths that contribute towards the exploration mission. Exploration tasks are divided into global exploration and local exploration. During global exploration, preferred paths include those with high volumetric gain in the grid map. Local exploration prioritizes sampling the immediate environment for new candidate graph nodes which are evaluated for connectivity to the main graph using candidate edges. The candidate edge is discretized into a series of candidate poses, and points spanning the vehicle footprint at each candidate pose are projected in the direction of gravity. Incident voxels are considered the “ground” and are tested for traversability and stair validity. A pose is:

• *traversability-valid* if the pose spans traversable voxels, i.e., the average traversability cost of the ground voxels is below an average traversability threshold (10% in this work) and the maximum traversability cost is below a maximum traversability threshold (20% in this work); and
• *stair-valid* if the pose spans voxels belonging to a stairway, i.e., a threshold (30% in this work) of the ground voxels have been classified as belonging to a stairway.

**FIGURE 6:**
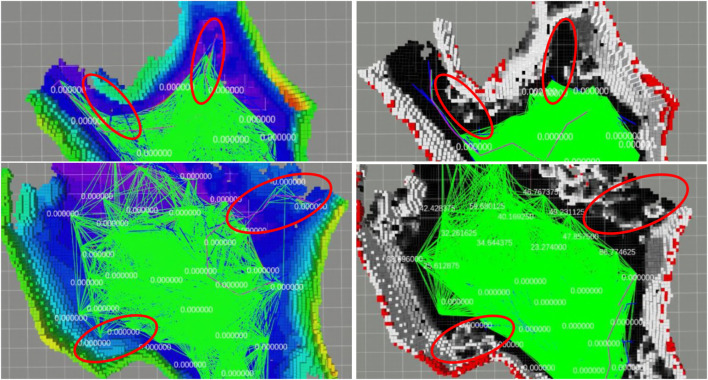
Qualitative simulation results of traversability-aware planning in the SubT Simulator’s *simple_cave_03*, a simulated cave environment. We compare the span of the planner graph (green) using the proposed traversability mapping method (right; where white voxels indicate non-traversable terrain, grey indicates semi-traversable, and black indicates traversable) to planning based simply on occupancy-only mapping (left; where the grid map is colored by height). Data was collected in two scenarios (top and bottom) from a single deployment. Highlighted (red circles) are instances where the planner’s graph (green lines) has extended into regions of rough terrain when planning without traversability information (left) but successfully avoided such regions when utilizing traversability information (right). An example of catastrophic navigation is also shown (rightmost red circle in the bottom-left panel) where the planner has chosen a path (purple line) that leads the robot into rough terrain, at which point the virtual robot becomes physically stuck.

Threshold values were carefully adjusted over extensive physical and simulated test deployments to ensure that the observed expansion of the planner graph (see Figures 6, 12) aligned with our objective of maintaining a conservative risk profile. If each pose along the candidate edge passes all relevant validity checks, then the candidate node and edge are added, thus expanding the planner graph.

To facilitate planning with both wheeled and quadrupedal robotic platforms, we use the following rules. The stair-validity test is ignored if the vehicle is not stair-capable. The traversability-validity test is ignored if the pose is stair-valid and the vehicle is configured to be stair-capable, due to the observation that when a voxel within the grid map is identified as part of a stairway (Section 2.3), it will often be perceived as untraversable (Section 2.2) as well.

If a stairway is mapped on a stair-capable robot, it will often result in the path planner directing the robot to traverse the stairway due to its associated volumetric gain and thus increased reward during global exploration. When a stairway is mapped for a stair-capable robot, the path planner is likely to optimize the robot’s trajectory to include traversing the stairway. This strategic decision is driven by the perceived volumetric gain associated with scaling the stairs, which contributes to an increased reward within the context of global exploration. In the case of the Boston Dynamics Spot robot, if the planner attempts to lead the Spot up a stairway, then prior to the start of the stairway, the low-level vehicle controller will activate Spot’s stair-optimized gait and switch from tracking the plan to tracking the straight path up the stairs described in Section 2.3.

To encourage cooperative exploration, we developed two “deconfliction” schemes. The first is *map deconfliction*, in which the vehicle performs global exploration using the merged map that consists of its own measurements as well as those that were measured by and received from other agents in the fleet. This allows the planner to waste no time generating paths to regions the robot has not seen previously but can result in less variety in trajectories. The second scheme is *pose deconfliction*, in which the planner performs global exploration using the historical trajectories of other agents in the fleet and is biased towards regions of the map that the fleet has not yet explored. This results in a greater variety of trajectories but can cause the vehicle to explore more slowly. The deconfliction schemes are not mutually exclusive and may also be run in parallel.

## 3 Results

We evaluate the proposed semantic grid mapping method in both simulated and physical experiments where robots autonomously explore previously unknown, mobility-challenged environments. In Section 3.1, we report on the traversability-mapping capabilities of the proposed method in a high-fidelity simulation environment. In Section 3.2, we physically demonstrate the utility of the stair-mapping capabilities of the proposed method. In Section 3.3, we examine the performance of the proposed mapping method as part of Team MARBLE’s semi-autonomous exploration system in the DARPA Subterranean Challenge (DARPA, 2022) where the system placed third.

### 3.1 Traversability simulation

Simulated environments provide an opportunity to quickly and repeatedly evaluate methods in a controlled environment. To this end, we utilize the DARPA SubT Simulator (OSRF, 2022; Chung et al., 2023) which was built to facilitate the development of and competition between robotic search-and-rescue software stacks. This physics-based simulator based on the Ignition Gazebo framework is open-sourced OSRF (2022) and includes simulated sensor noise, realistic terramechanics, and bandwidth-constrained RF communications, as well as several worlds which emulate subterranean environments, such as subway tunnels and caves.

We qualitatively evaluate and illustrate in Figure 6 the effect of the traversability-mapping capability described in Section 2.2 on the propagation of the corresponding planner graph described in Section 2.5. Data was collected by running our terrain-aware navigation stack in two scenarios in the *cave_simple_03* virtual world of the SubT Simulator: with traversability-mapping capabilities enabled, and with occupancy-only mapping capabilities. Graph extension is used as a qualitative metric in each scenario to compare planning performance with traversability information to that which only utilizes occupancy information. Several instances are highlighted in which the planner graph incorrectly extended into regions of rough terrain as a result of only utilizing occupancy information, but then avoided extending into such regions when traversability was considered.

**FIGURE 7:**
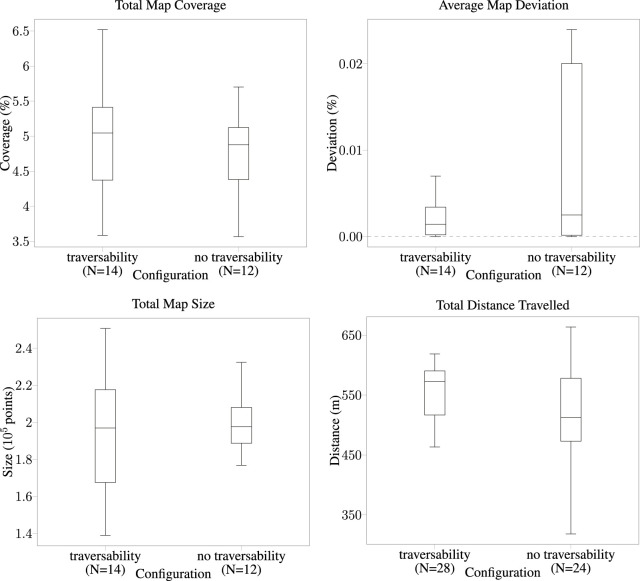
Quantitative simulation results for cooperative exploration performance in the SubT Simulator’s *simple_cave_01*, a simulated cave environment. We compare exploration performance using the proposed traversability mapping method (“traversability”) to occupancy-only mapping (“no traversability”). Data was collected from twenty-six deployments of a fleet of two Husky robots.

We also evaluate the effect of the traversability-mapping capability quantitatively via twenty-six simulated deployments of a fleet consisting of two Husky robots in the SubT Simulator (Figure 7). Robots were simply tasked with autonomously exploring the *cave_simple_01* virtual world of the SubT Simulator. We operated the proposed mapping system on both robots simultaneously during 1-h deployments and varied whether the traversability-mapping capabilities were enabled or disabled prior to starting. On simulation start, the first Husky was programmed to immediately start exploring; the second Husky was programmed to start exploring 1 min later. In all deployments, robots operated with occupancy- and stairway-mapping capabilities enabled; in 54% of deployments, robots also operated with traversability-mapping capabilities enabled (“traversability”). *Pose deconfliction* and *map deconfliction* (as explained in Section 2.5) were enabled and disabled, respectively, for all deployments. This is the same cooperation strategy employed during the SubT Final Event (as described in Section 3.3) and allows for a meaningful comparison between simulated and physical experiments. Simulated deployments were performed on a PC running Ubuntu 18.04 with an AMD Ryzen 9 5900X 12-Core processor and an NVIDIA GeForce RTX 3070.

Performance is evaluated via several metrics:

1) *Total map coverage*, the percentage of ground-truth points near (within a threshold distance) an input map point at the end of the run;
2) *Average map deviation*, the average percentage of input map points far (greater than a threshold distance) from the nearest ground-truth point over the run;
3) *Total map size*, the number of points in the input map at the end of the run; and
4) *Total distance traveled* the total path length of each vehicle’s trajectory at the end of the run.

Metrics one to three are provided by the SubT Map Analysis package (Schang et al., 2021) and apply to the performance of the whole fleet. Metric 4 applies to the performance of each robot, hence the doubling in the number of trials.

### 3.2 Stair demonstration

**FIGURE 8:**
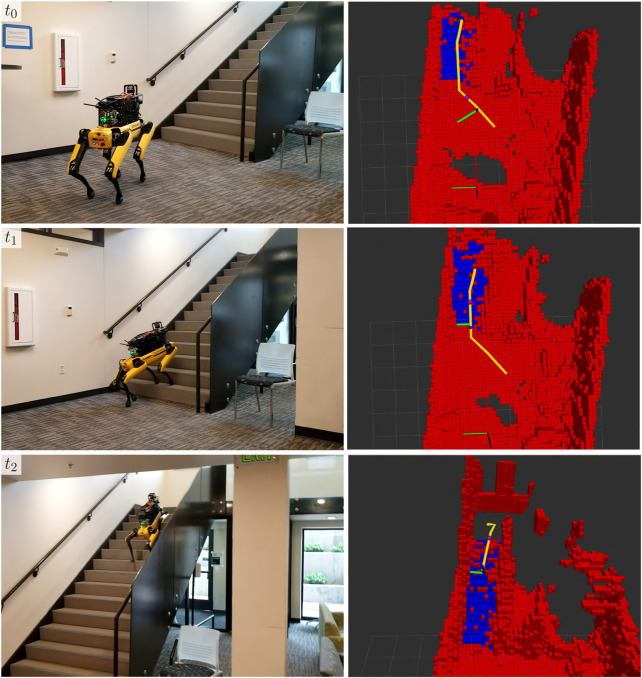
A demonstration of stair-aware planning where a Spot robot traverses a stairway (left) in the Engineering Center at the University of Colorado Boulder in Boulder, CO, United States of America. The corresponding semantic grid map and the pose of the robot with respect to it are shown (right), with stair voxels shown in blue.

We evaluate the stair-mapping and planning capabilities in real-world scenarios using the Boston Dynamics Spot platform. Due to limitations in simulated controllers for quadruped robots in the SubT Simulator, we were unable to quantitatively evaluate stair traversal by a quadruped. However, we observe that across hundreds of hours of physical testing leading up to the SubT Final Event, the Spot was successfully able to plan up all encountered stairways. A qualitative demonstration of the Spot planning up a stairway at the University of Colorado Boulder Engineering Center is shown in Figure 8. Note that our implementation of the proposed method is limited to only planning new routes over ascending stairways, due to constraints on the field-of-view of the lidar sensor.

### 3.3 DARPA subterranean (SubT) challenge

DARPA organized the SubT Challenge, a series of four competition events held between August 2018 and September 2021, to motivate the development of new robot technologies. The challenge involved teams from academic and industrial backgrounds who were tasked with designing multi-agent robotic exploration systems dedicated to search and rescue operations (DARPA, 2022). These teams competed in a variety of subterranean environments, including subterranean mines, urban areas like subway tunnels, and cave-like structures. The primary objective was to successfully navigate and search these complex terrains in order to locate artifacts, which were pre-defined objects used as indicators of human presence.

**FIGURE 9:**
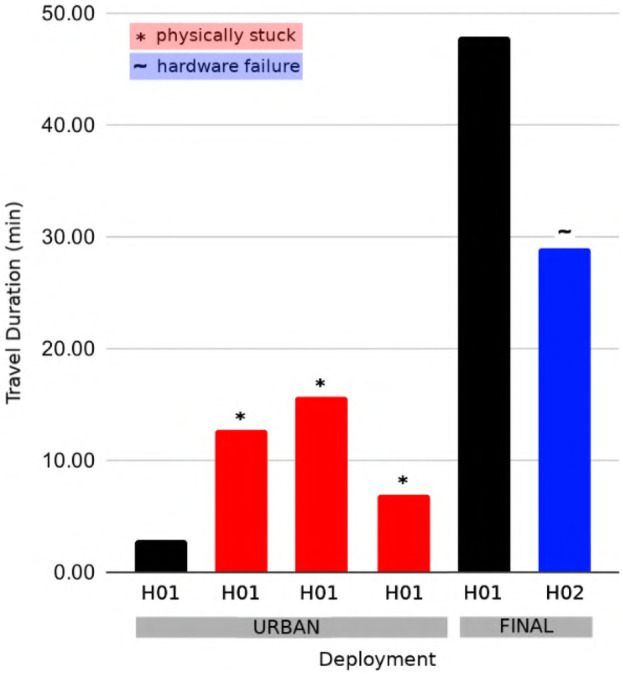
Travel duration is illustrated for physical competition deployments of wheeled Husky robots at the SubT Circuit Urban Event and the SubT Final Circuit Event. Deployments that were unable to explore for the total hour due to becoming stuck or experiencing a hardware failure are indicated. Note that the terrain-aware semantic grid mapping method proposed in this work was introduced between the Urban and Final events.

We compare the travel duration of robots from the Urban Circuit Event and the Final Circuit Event of the SubT Challenge to evaluate the performance of the traversability mapping system. In the urban event, we approximated the traversability of the terrain based solely on the occupancy grid map and utilized a Frontier-based navigation system (Ohradzansky et al., 2020; Ohradzansky et al., 2021). We developed the traversability mapping component of the mapping framework proposed in this work and deployed it in the final event to improve the travel duration and thus exploration capabilities of the robots in the fleet. We observe that the proposed mapping framework approximately doubled the total exploration time across wheeled robots in realistic scenarios filled with complex terrain (Figure 9). Furthermore, none of the robots running the traversability mapping component became physically stuck during the final event.

**FIGURE 10:**
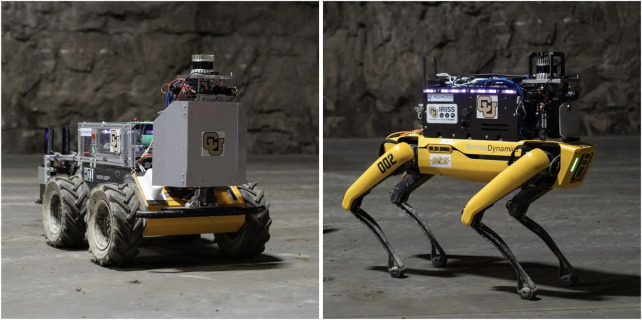
The Clearpath Husky (left) and Boston Dynamics Spot (right) robots used in physical testing and competition in the SubT Final Event.

Team MARBLE fielded two types of robots in the SubT Challenge Final Circuit Event (Figure 10): two differential drive ClearPath Husky A200 robots (denoted with the name prefix “H”), and two quadrupedal Boston Dynamics Spot robots (denoted with the name prefix “D”). The Husky robots utilized a 64-core AMD Ryzen Threadripper 3990X equipped with 128 GB of RAM, 4 TB of SSD storage, and dual NVIDIA GTX 1650 GPUs to accelerate object detection inference times. The Spot robots utilized an AMD Ryzen 5800U with 64 GB of RAM and 2 TB of storage. This main computer was paired with a Jetson Xavier AGX to process the camera streams and perform object detection. Both robots also featured Ouster OS-1 64-beam lidars for localization and mapping, a LORD Microstrain 3DM-GX5-15 IMU for localization, and several pieces of commercial and custom electrical hardware for battery management and power distribution.

In the implementation of the terrain-aware navigation stack used in the final event, only the Husky robots utilized the traversability information in the map, as the rough terrain avoidance capabilities built-in to the Spot robot were deemed capable of ensuring robot survival. This decision stemmed from the adoption of a strategy that discouraged any inhibition of the Spot robots’ ability to explore. Additionally, only the Spot robots were deemed stair-capable and thus used the stair-presence information to explore and traverse stairs. Finally, to conserve network bandwidth, only *pose deconfliction* as described in Section 2.5 was used.

**FIGURE 11:**
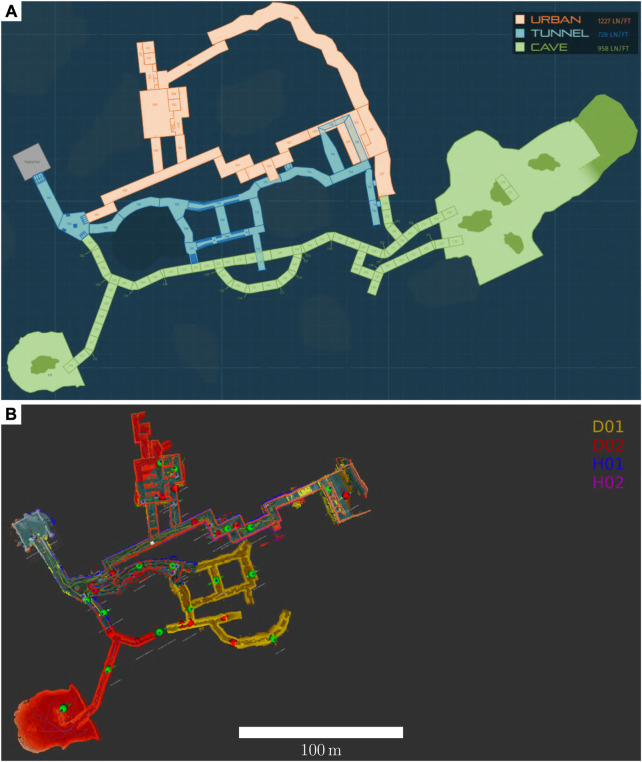
Merged map **(B)** from four robot agents during their deployment in the Subterranean Challenge Final Event course **(A)**. The map colors in A indicate the three types of subterranean environments which were emulated: tunnel, urban, and cave. In B, yellow and red maps indicate those from quadruped Spot robots, and blue and pink maps indicate those from wheeled Husky robots. All robots were deployed from the square room on the left side of the map, shown in grey in **(A)**. This map has been corrected for localization errors to illustrate the collective mapping capabilities of MARBLE Mapping.

During the final event, *diff maps* from all robots are transmitted to the base station and a merged map was constructed to provide situational awareness and enable flexible, semi-autonomous control to a human operator. The merged map (Figure 11) illustrates the extent to which the fleet explored the environment.

**FIGURE 12:**
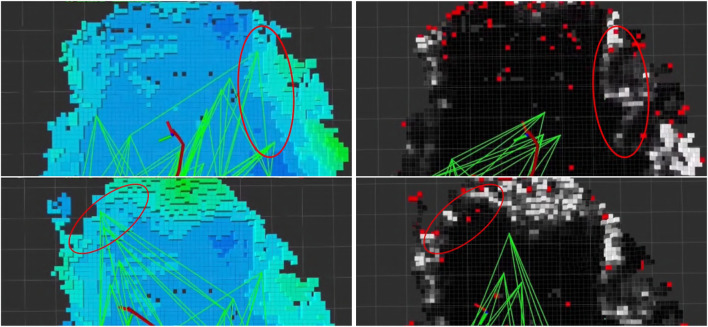
Results of traversability-aware planning (right; where white indicates non-traversable, grey indicates semi-traversable, and black indicates traversable) compared to planning based simply on occupancy (left; where the grid map is colored by height). Data was collected from a single deployment for two scenarios (top and bottom) in a physical subterranean environment. Highlighted (red circles) are instances where the planner’s graph (green lines) has extended into regions of rough terrain when planning without traversability information but successfully avoided such regions when utilizing traversability information.

We illustrate the qualitative performance of the traversability-mapping capabilities in physical environments (Figure 12) using the approach introduced in Section 3.1. Planner graph extension is used as a metric to compare planning performance with traversability information to that which only utilizes occupancy information. Data was collected by running Team MARBLE’s terrain-aware navigation stack during a post-competition deployment at the SubT Final Circuit Event.

**FIGURE 13:**
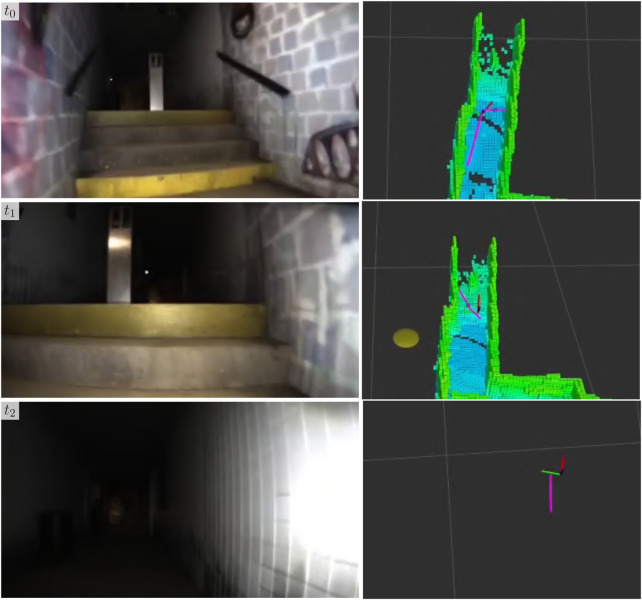
Example of stair traversal by a Boston Dynamics Spot robot during the SubT Final Circuit Event competition. The forward-facing camera of the robot (left) and the corresponding robot pose (right) is shown. In the top two rows, the occupancy map and current path are shown. In the bottom row, the occupancy map has been hidden to visualize the straight-line path produced by the stair-mapping module for a low-level vehicle controller, as described in Section 2.3.

Qualitative results of the stairway classification and mapping method developed in Section 2.3 at the final event are shown in Figure 13, in which a Spot robot traverses a short flight of stairs in the urban portion of the environment during a competition deployment. The planner directs the robot up the stairs, however, because it accurately detects the stairs and extracts the straight-line path up the stairs, it follows the straight-line path rather than the original plan which would have led the robot up the stairs at an angle and jeopardized the deployment. As a result of traversing this stairway, the robot was able to detect one more artifact, and potentially two more had the object detection stack worked as expected. Since it had already seen the stairway, the robot was also able to easily plan back down the stairway and continue exploring other areas of the map.

## 4 Discussion

### 4.1 Performance of traversability-mapping

The traversability-mapping component of the proposed terrain-mapping solution performed excellently in its function of facilitating safe and conservative planning in order to enable continuous exploration. This is evidenced by the improvement in travel duration upon introduction of the proposed terrain-mapping solution as shown in Figure 9. As can be seen, the robots which utilized the traversability-aware navigation stack in the final event traveled for much longer, and without any instances where they got physically stuck. This is largely a result of the traversability mapping capabilities of the terrain-aware mapping method presented in this chapter, however, the roles of operational experience and other technical improvements in maximizing exploration duration cannot be ignored. The qualitative effects are apparent when comparing the planning graph for traversability mapping to occupancy-only mapping (Figures 6, 12). An example of catastrophic navigation is shown in Figure 6, where the occupancy-only planner directs the robot to enter rough terrain, at which point the robot becomes physically stuck and the simulated deployment must terminate. The quantitative effects are also significant, as shown in Figure 7. We see that traversability-aware mapping results in more distance traveled due to enduring exploration and less average map deviation due to reduced localization error caused by traversing rough terrain. While traversability-mapping also enabled the greatest map coverage and largest map size, the aggregate improvement is less significant, likely due to the deconfliction strategy discussed in Section 2.5.

### 4.2 Performance of stair-mapping

The stair-mapping component of the proposed terrain-mapping solution performed well in its function of facilitating more expressive exploration. This is evidenced by the additional artifact that the robot scored as a result of climbing a short flight of stairs during the final event competition deployment. The effect of this artifact is particularly fortunate considering that Team MARBLE scored just one point higher than the next-highest scorer DARPA 2022.

### 4.3 Limitations

Despite the excellent performance, the proposed terrain-mapping system has some limitations.

Perhaps the most crucial limitation is the challenge of observing and subsequently mapping and traversing a descending stairway, due to the lidar sensor lacking a field of view that encompasses the ground directly in front of the robot. This limitation resulted in the vehicle failing to explore a large region of the environment in the SubT Final Event.

Another limitation is the inability to reliably map very thin obstacles, such as dangling cables or wire fencing which feature prominently in true search and rescue scenarios. This challenge is inherent to many grid mapping approaches and requires more sophisticated classification techniques to address.

**FIGURE 14:**
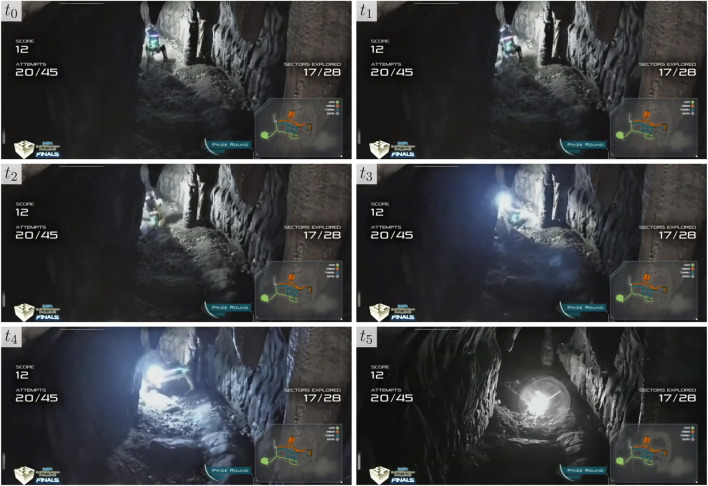
Instance from the SubT Final Event where a Spot robot became unstable while attempting to traverse rough terrain. The robot was not running the traversability mapping component proposed in this work. Photo credit: DARPA.

Finally, as described in Section 3.3, the Spot robots operated in the SubT Final Event without the proposed traversability-mapping component to maximize exploration. While this may have improved the Spot robot exploration performance, it also resulted in one instance where the robot attempted and failed to traverse rough terrain, resulting in a fall and no subsequent travel for the remainder of the run (Figure 14).

### 4.4 Conclusions and future work

Future improvements may address these aforementioned limitations and enable even safer and more comprehensive exploration. One such improvement would be to expand the field of view of the stairway classification and mapping sensors to enable the traversal of descending stairways. Future work may also include the “roughness” (i.e., the quality of a plane-fitting operation) in the traversability cost calculation. This would improve the perception of thin obstacles and the traversability classification of rough terrain, however, such operations can be computationally expensive. Finally, the proposed mapping framework’s modularity facilitates its compatibility with various multi-agent and navigation stacks. Future work may utilize this feature to allow an independent evaluation of its performance.

Nonetheless, the proposed terrain-mapping solution demonstrates an effective method to enable safe and enduring exploration in large, unstructured, subterranean environments with bandwidth-constrained communication. This is evidenced by statistical analysis of performance in simulation and Team MARBLE’s third place finish in the DARPA Subterranean Challenge Final Event.

## Data Availability

The datasets presented in this study can be found in online repositories. The names of the repository/repositories and accession numbers can be found below: arpg.colorado.edu.
